# A multilevel analysis of the determinants of HIV testing among men in Sub-Saharan Africa: Evidence from Demographic and Health Surveys across 10 African countries

**DOI:** 10.1371/journal.pgph.0003159

**Published:** 2024-05-02

**Authors:** Mukhtar A. Ijaiya, Adedotun Anibi, Mustapha Muhammed Abubakar, Chris Obanubi, Seun Anjorin, Olalekan A. Uthman

**Affiliations:** 1 Jhpiego, Abuja, Federal Capital Territory, Nigeria; 2 District Health Unit, Kawartha, Ontario, Canada; 3 Directorate of Therapeutic Services, Medical Services Branch, Nigerian Air Force, Abuja, Federal Capital Territory, Nigeria; 4 School of Biodiversity, One Health and Veterinary Medicine, College of Medical, Veterinary and Life Sciences, University of Glasgow, Glasgow, United Kingdom; 5 United States Agency for International Development, Gaborone, Botswana; 6 Nuffield Department of Population Health, Big Data Institute, University of Oxford, Oxford, United Kingdom; 7 Division of Health Sciences, Warwick Centre for Global Health, Warwick Medical School, University of Warwick, Coventry, United Kingdom; Conservatoire national des arts et metiers, FRANCE

## Abstract

Sub-Saharan Africa, the epicenter of the HIV epidemic, has seen significant reductions in new infections over the last decade. Although most new infections have been reported among women, particularly adolescent girls, men are still disadvantaged in accessing HIV testing, care, and treatment services. Globally, men have relatively poorer HIV testing, care, and treatment indices when compared with women. Gender norms and the associated concept of masculinity, strength, and stereotypes have been highlighted as hindering men’s acceptance of HIV counseling and testing. Therefore, men’s suboptimal uptake of HIV testing services will continue limiting efforts to achieve HIV epidemic control. Thus, this study aimed to identify individual, neighborhood, and country-level determinants of sub-optimal HIV testing among men in Sub-Saharan African countries. We analyzed demographic and health datasets from surveys conducted between 2016 and 2020 in Sub-Saharan African Countries. We conducted multivariable multilevel regression analysis on 52,641 men aged 15–49 years resident in 4,587 clusters across 10 countries. The primary outcome variable was ever tested for HIV. HIV testing services uptake among men in these ten Sub-Saharan African countries was 35.1%, with a high of 65.5% in Rwanda to a low of 10.2% in Guinea. HIV testing services uptake was more likely in men with increasing age, some form of formal education, in employment, ever married, and residents in relatively wealthier households. We also found that men who possessed health insurance, had some form of weekly media exposure, and had accessed the internet were more likely to have ever received an HIV test. Unlike those noted to be less likely to have ever received an HIV test if they had discriminatory attitudes towards HIV, comprehensive HIV knowledge, recent sexual activity, and risky sexual behavior were positive predictors of HIV testing services uptake among men. Furthermore, men in communities with high rurality and illiteracy were less likely to receive an HIV test. Individual and community-level factors influence the uptake of HIV testing among Sub-Saharan African men. There was evidence of geographical clustering in HIV testing uptake among men at the community level, with about two-thirds of the variability attributable to community-level factors. Therefore, HIV testing programs will need to design interventions that ensure equal access to HIV testing services informed by neighborhood socioeconomic conditions, peculiarities, and contexts.

## Introduction

In 2021, there were approximately 38.4 million persons living with human immunodeficiency virus (HIV) around the world, with about 1.5 million new infections and 650,000 deaths from HIV-related illnesses in the same year [1, 2]. Of this number, only about 85% knew their HIV status, with 88% accessing care and 92% virally suppressed [2]. These proportions fall short of reaching the global targets of 95-95-95 aimed at ending AIDS as a public health threat by 2030 [3]. Key to achieving these goals has been the recognition of the imperative of addressing inequalities and disparities in accessing HIV testing, care, and treatment services across socioeconomic and demographic groupings [1, 3].

Sub-Saharan Africa, the epicenter of the HIV epidemic, has seen significant reductions in new infections over the last decade [1, 4]. However, Sub-Saharan Africa still accounts for about 59% of new HIV infections and 67% of People Living with HIV (PLHIV) [1, 2, 4, 5]. In addition, although most new infections have been reported among women, particularly adolescent girls, men are still disadvantaged in accessing HIV testing, care, and treatment services [1, 6]. Globally, men have relatively poorer HIV testing, care, and treatment indices when compared with women, with an estimated 18% of men living with HIV who do not know their status compared to 12% among women [1, 2]. Of the HIV-positive men who knew their status, 32% were not on antiretroviral treatment compared to 21% of women [1, 2]. Furthermore, 38% of HIV-positive men on antiretrovirals had not achieved viral suppression, in contrast to 28% of women [1, 2]. HIV testing is the crucial entry point in the 95-95-95% cascade and is often the main impediment to engaging men in the continuum of HIV care and treatment [3].

Generally, men’s poor health-seeking behavior has been noted in several studies [7, 8]. Gender norms and the associated concepts of masculinity, strength, and stereotypes have been highlighted as hindrances to men’s acceptance of HIV counseling and testing [9–11]. In addition, gendered health facilities, health policies, service delivery practices, stigma, and discrimination have also been posited as critical challenges to men’s access to HIV counseling and testing [10, 12, 13]. Therefore, men’s suboptimal uptake of HIV counseling and testing (HCT) will continue to limit efforts at achieving HIV epidemic control [1, 4, 6, 9, 14].

Studies have found that the limited uptake of HIV testing among men is associated with poor knowledge of service availability, lack of health insurance coverage, sexual behavior, and low-risk perceptions [15, 16]. Magadi and Desta, 2011 investigated the general patterns and risk factors of HIV seropositivity across countries in Sub-Saharan Africa and found that for both males and females, the risk of being HIV seropositive was relatively higher among urban residents, those in middle or wealthier households, and those who are not circumcised [17]. Several studies have also shown that age, better HIV knowledge, condom use, knowledge of partner’s HIV status, higher income and higher education, internet usage, mass media, and socioeconomic status are positively associated with men’s likelihood of accepting to be tested for HIV [13, 16, 18–23].

Several interventions have been mooted to improve men’s uptake of HIV testing. These include workplace-based HIV testing services, HIV self-testing, and couple-oriented HIV counseling [11, 24, 25]. However, a need remains to better understand the drivers and determinants of men’s uptake of HIV testing services toward designing effective interventions [13, 16]. Most of the literature reviewed predominantly centered on the factors influencing HIV testing at the individual level [13–16, 19, 21, 23, 25]. However, this approach may inaccurately attribute correlations observed at the individual level to broader groups or erroneously apply associations found in group-level data to individuals [26]. Therefore, the objective of this study was to examine individual, neighborhood, and country-level factors as well as an in-depth analysis of the patterns and predictors of geographical and social disparities of HIV testing uptake among men in Sub-Saharan Africa.

## Methods

### Study design and data sources

The men’s data-male recode component of the most recent Demographic and Health Survey (DHS) round 7 or 8 datasets, which has a record of every eligible man interviewed as determined by the household schedule, was used for this analysis. ICF International conducts these surveys in low- and middle-income countries (LMIC) [27]. DHS datasets provide highly recognized gold-standard vital information on nationally representative household surveys from over 90 LMICs [27]. The DHS program collects cross-sectional data using standard model questionnaires, modified in the eight phases since the survey implementation began [27]. DHS surveys provide country-wide data on family planning, nutrition, reproductive health, and various healthcare areas in each implementing country every five years [27]. Countries are encouraged to adopt the questionnaire, but questions of interest can be added, and questions that do not pertain to the country may be deleted [27]. The survey data is collected from men aged between 15–49, 15–54, or 15–59, and women aged between 15–49, and their under-five-year-old children in randomly selected households using a stratified multistage cluster sampling design [27].

### Outcome variable

In this study, we defined the outcome variable, HIV testing, at the individual level. HIV testing was defined as males who indicated to have once taken an HIV test (ever taken an HIV test). The DHS variable "ever been tested for HIV?" was used.

### Individual level variables

The individual-level male-specific factors included in this analysis were age, education, wealth index constructed by DHS, occupation, marital status, internet usage, health insurance coverage, and recent sexual activity [27, 28]. We also included the DHS indicators as defined by the DHS guide: exposure to mass media, comprehensive knowledge about HIV, and discriminatory attitudes towards people living with HIV [27, 29, 30]. Risky sexual behavior was constructed from recoding responses of those who had sexual intercourse in the 12 months preceding the survey with a person who was neither their spouse nor lived with them and did not use a condom the last time they had sexual intercourse with a person who was neither their spouse nor lived with them [27]. The conceptual framework for HIV testing by Lakhe et al. 2020 and availability informed variable selection [31].

### Neighborhood variables

Neighborhoods, also called clusters/communities, are the Primary Sampling Unit (PSU) for DHS data collection. Neighborhoods are defined as a cluster of respondents living in households of proximity and are typically census enumeration areas. The neighborhoods in this dataset are stratified and chosen with probability proportional to size within each stratum, with the systematic sampling of households adequate for intra- and inter-community analysis similar to previous studies [32, 33]. The community-level variables selected are illiteracy, unemployment rate, poverty level, and community rurality. These were recategorized from the individual level variables, education, occupation, wealth index, and residence, respectively, into low and high, using the median value across all communities for each variable as the benchmark for grouping.

### Country level variables

Human Development Index (HDI), Domestic General Government Health Expenditure as a percentage of gross domestic product (GDP) (%) (GGHE-D), and HIV prevalence retrieved from the Human Development Report and The Global Health Observatory, respectively, were the country level variables included in this model [34, 35]. According to the Human Development Report, HDI is a compound measure of life expectancy, income per capita, and education. The variable was categorized into low and medium based on the HDI fixed cut-off points [34]. GGHE-D, categorized into tertiles (low, medium, and high), details public spending on health from domestic sources as a proportion of the economy [36]. The HIV prevalence data highlights the relative HIV prevalence burden across countries, divided into tertiles: low, medium, and high.

### Statistical analyses

The analysis was adjusted for sample weight, clustering, and stratification. Univariate descriptive analysis was conducted with responses reported as absolute numbers (percentages) for categorical variables and mean (Standard Deviation S.D.) for continuous variables. Furthermore, country, neighborhood, and individual level factors associated with HIV testing amongst men were analyzed using multivariate multilevel logistics regression (MLRA), with individual male characteristics (as level 1), living in a neighborhood (as level 2), and in a country (as level 3). The disparities in HIV uptake among men can best be explained using MLRA [37–40]. MLRA is the statistical method of choice in describing the magnitude of neighborhood clustering on individual health and distribution between and across individual and neighborhood levels [37–40]. Moreover, MLRA allows for quantifying the effect of the association between individual determinants, neighborhood composition, and country characteristics [37–40].

Five models were built: a null model with no explanatory factors to ascertain the variation in the likelihood of HIV testing between communities and countries. Models 2, 3, and 4 only included variables at the individual level, neighborhood level, and country level, respectively. Model 5 had all variables at the individual, community, and country levels.

The associations between the variables (fixed effects) were reported using odds ratio (OR) at a 95% credible interval (Crl). Median Odds Ratio (MOR) and Intraclass Correlation Coefficient (ICC) were used to estimate the variance (random effects). MOR is an estimation of the variance attributable to neighborhood and country effects, and this variance is directly proportional to rising MOR, with an MOR of one equaling no community or country variance [37, 38, 40, 41]. At the same time, ICC measures the similarity amongst study participants living in the same neighborhood and same country [37, 38, 40, 41].

STATA17 S.E. was used for the descriptive analyses, and the MLwinN software, version 3.05 in STATA17 S.E., was used to fit the multilevel models [42, 43]. We used the Bayesian Markov Chain Monte Carlo process in this multilevel analysis, which produces unbiased estimates and superior properties [44, 45]. To evaluate model fit, we used the Bayesian Deviance Information Criterion [44].

### Ethics statement

This study is based on a secondary dataset from the DHS; therefore, ethical approval was not required. We obtained permission from the MEASURE DHS project to download and use the datasets.

## Results

### Characteristics of the study population

This study included a total of 52,641 men between the ages of 15–49. They were residents in 4,587 communities across 10 Sub-Saharan African countries, namely Angola, Benin, Burundi, Cameroon, Gambia, Guinea, Liberia, Mali, Rwanda, and Sierra Leone. These 10 DHS-7 and 8 country datasets with all the questions and variables of interest were selected from the 15 available country datasets at the time of download. The included surveys were conducted between 2016 and 2020 (Table 1). Angola had the highest number of communities (625), and Gambia had the least number of communities (280); the median number of communities was 465. Six countries included in the analysis were in the West African region, and the remaining four were evenly split between the Eastern and Middle African regions.

**Table 1:** Description of Demographic and Health Surveys data by countries.

Sub-region	Country	Survey Year	Number of Men	Number of Neighbourhoods	Ever Tested for HIV (%)	Human Development Index (HDI)	GGHE-D (%)	HIV Prevalence (%)
Middle Africa	Angola	2016	5,377	625	33.65	Medium HDI	Second Tertile	Second Tertile (1.8)
Western Africa	Benin	2018	6,731	555	19.99	Low HDI	First Tertile (Low)	First Tertile (1.0)
Eastern Africa	Burundi	2017	6,697	554	49.09	Low HDI	Third Tertile (High)	First Tertile (1.1)
Middle Africa	Cameroon	2019	6,063	429	56.62	Medium HDI	First Tertile (Low)	Third Tertile (3.1)
Western Africa	Gambia	2020	4,201	280	26.80	Low HDI	First Tertile (Low)	Second Tertile (1.8)
Western Africa	Guinea	2018	3,577	400	10.16	Low HDI	Third Tertile (High)	Second Tertile (1.4)
Western Africa	Liberia	2020	3,760	325	33.66	Low HDI	First Tertile (Low)	First Tertile (1.2)
Western Africa	Mali	2018	4,037	345	14.39	Low HDI	Second Tertile	First Tertile (0.9)
Eastern Africa	Rwanda	2020	5,833	500	65.50	Low HDI	Third Tertile (High)	Third Tertile (2.6)
Western Africa	Sierra Leone	2019	6,365	574	22.87	Low HDI	Second Tertile	Second Tertile (1.6)

GGHE-D: Domestic General Government Health Expenditure as a percentage of gross domestic product (GDP)

Consequently, the West Africa region accounts for just above half (54%) of the study population, with Eastern and Middle Africa having 24% and 22% of the study population, respectively. Only two countries (Angola and Cameroon) were in the medium HDI category, with the rest in the low HDI category. Of the countries included in this study, two were in the high HIV prevalence tertile, and four each were in the medium and low HIV prevalence tertile, respectively. The GGHE-D tertiles had four countries in the low category and three countries each in the medium and high categories, respectively.

**Fig 1:**
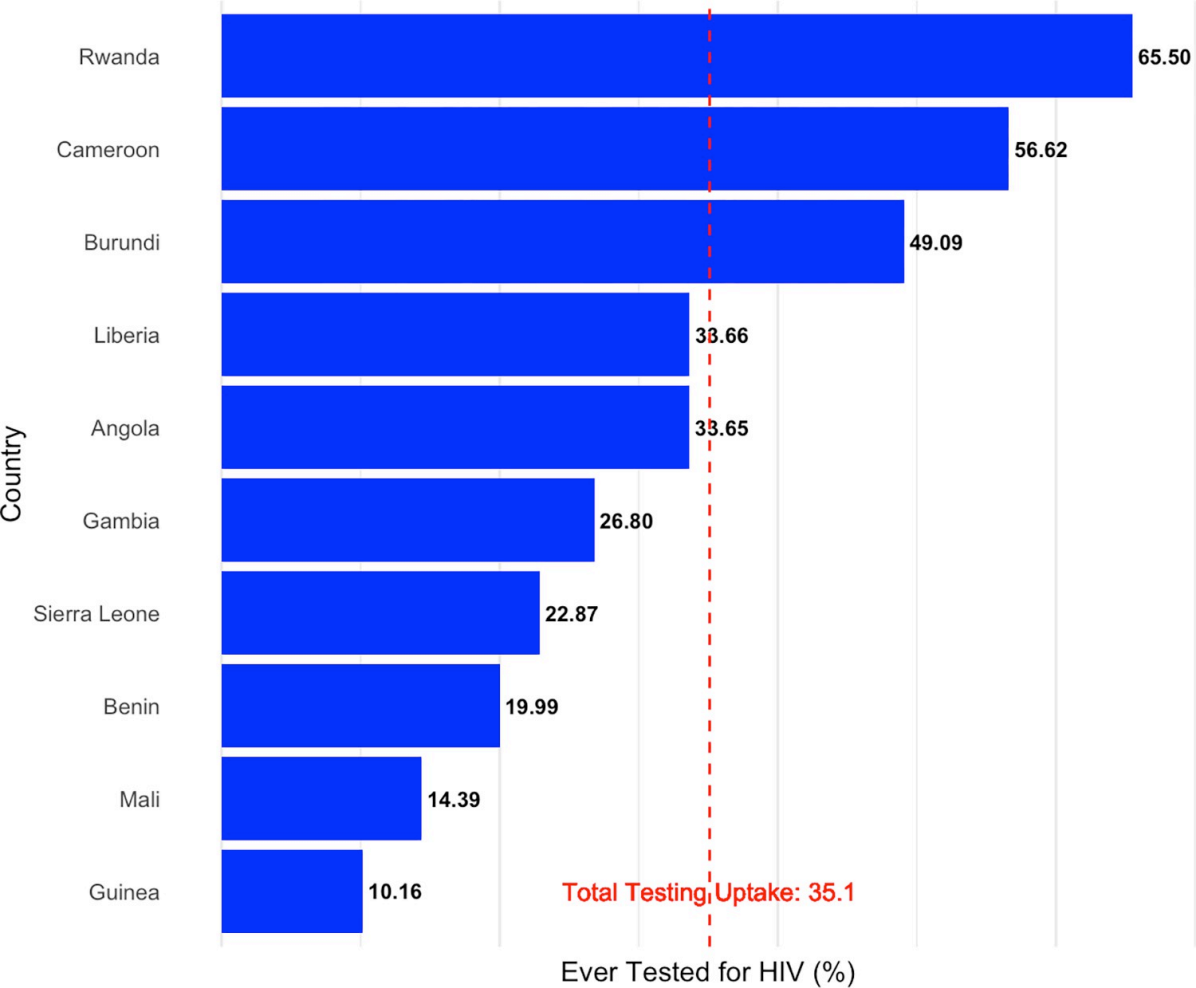
Percentage of men ever tested for HIV by countries.

More than one-third (35.1%) of the men had ever been tested for HIV. Rwanda had the highest proportion (65.5%) of men who had received an HIV test, followed by Cameroon (56.6%) (Fig 1). All the other eight countries had testing percentages lower than 50%, with the least proportion of men ever tested for HIV in Guinea (10.2%). Table 2 shows the pooled sample characteristics of the 10 countries included in the analysis. The mean age among these male respondents was 29 years, and more than two-thirds (77%) had ever had sexual intercourse. A quarter (25.1%) were residents in the wealthiest wealth index households, and only 16.4% were residents in the poorest households. There was an almost equal proportion of those who never married (47.7%) and married or lived together (49.7%) and those with discriminatory attitudes (49.8%) and non-discriminatory attitudes (50.2%) to HIV. The majority of the men were currently in employment (83%), had some form of education (76.3%), and had no health insurance (84.7%). Most of the men had never used the internet (65.0%), had weekly exposure to a form of mass media (63.7%), had comprehensive HIV knowledge (65.7%), and did not have any risky sexual behavior (71.8%). Community rurality was 9.7%, illiteracy was 44.8%, poverty was 41.7%, and unemployment was 46.7%.

**Table 2:** Summary of the 10 countries pooled sample characteristics of DHS data.

Variables	Overall Number(%)	Never tested for HIV Number(%)	Ever tested for HIV Number(%)	P-Value
**Total (N)**	52,641	34,325 (64.9)	18,583 (35.1)	
**Age (in years) Mean (SD)**	28.5 (9.9)	27.0 (10.1)	31.3 (8.9)	< .001** **
**Education (Number(%))**		** **		<0.001
No Education	12,510 (23.7)	10,183 (29.7)	2,327 (12.5)	
Primary	14,931 (28.2)	9,371 (27.3)	5,560 (29.9)	
Secondary	21,425 (40.5)	13,365 (38.9)	8,060 (43.4)	
Higher	4,041 (7.6)	1,405 (4.1)	2,635 (14.2)	
**Wealth Index (Number(%))**				<0.001
Poorest	8,692 (16.4)	6,721 (19.6)	1,970 (10.6)	
Poorer	9,511 (18.0)	6,794 (19.8)	2,716 (14.6)	
Middle	10,242 (19.4)	6,767 (19.7)	3,475 (18.7)	
Richer	11,164 (21.1)	6,944 (20.2)	4,220 (22.7)	
Richest	13,298 (25.1)	7,097 (20.7)	6,201 (33.4)	
**Occupation (Number(%))**				<0.001
Working	44,028 (83.3)	27,438 (80.0)	16,590 (89.3)	
**Marital Status (Number(%))**				<0.001
Never Married	25,225 (47.7)	18,679 (54.4)	6,546 (35.2)	
Married/Living together	26,269 (49.7)	14,863 (43.3)	11,406 (61.4)	
Divorced/Separated/Widowed	1,414 (2.7)	783 (2.3)	630 (3.4)	
**Internet Usage (Number(%))**				<0.001
Never	34,419 (65.0)	24,217 (70.6)	10,201 (54.9)	
Yes, last 12 months	17,017 (32.2)	9,240 (26.9)	7,777 (41.9)	
Yes, before last 12 months	1,472 (2.8)	868 (2.5)	604 (3.3)	
**Health Insurance Coverage (Number(%))**				<0.001
Yes	8,097 (15.3)	2,810 (8.2)	5,288 (28.5)	
**Recent Sexual activity (Number(%))**				<0.001
Never had sex	12,050 (22.8)	9,928 (28.9)	2,122 (11.4)	
Active in last 4 weeks	27,076 (51.2)	15,543 (45.3)	11,533 (62.1)	
Not active in last 4 weeks	13,782 (26.0)	8,854 (25.8)	4,928 (26.5)	
**Exposure to Mass Media (Number(%))**				<0.001
Weekly access to at least one media	33,697 (63.7)	19,957 (58.1)	13,740 (74.0)	
**Discriminatory Attitudes (Number(%))**				<0.001
Discriminatory Attitudes	25,137 (49.8)	19,369 (60.8)	5,768 (31.0)	
**Comprehensive HIV Knowledge (Number(%))**				<0.001
Comprehensive HIV Knowledge	34,769 (65.7)	19,549 (57.0)	15,220 (81.9)	
**Risky Sexual Behaviour (Number(%))**				<0.001
Risky sexual behavior	14,919 (28.2)	9,258 (27.0)	5,660 (30.5)	
**Community-level Poverty (Number(%))**	22,073 (41.7)	15,834 (46.1)	6,240 (33.6)	<0.001
**Community-level Illiteracy (Number(%))**	23,698 (44.8)	16,674 (48.6)	7,024 (37.8)	<0.001
**Community-Level Unemployment (Number(%))**	24,717 (46.7)	15,785 (46.0)	8,932 (48.1)	0.0282
**Community-level Rurality (Number(%))**	5,130 (9.7)	3,551 (10.4)	1,579 (8.5)	<0.001
**Human Development Index (Number(%))**				<0.001
Low	41,361 (78.2)	28,070 (81.8)	13,290 (71.5)	
Medium	11,547 (21.8)	6,255 (18.2)	5,293 (28.5)	
**HIV Prevalence (Number(%))**				<0.001
First Tertile (Low)	21,264 (40.2)	14,770 (43.0)	6,494 (34.9)	
Second Tertile (Medium)	19,672 (37.2)	14,881 (43.4)	4,792 (25.8)	
Third Tertile (High)	11,972 (22.6)	4,674 (13.6)	7,298 (39.3)	
**GGHE-D (Number(%))**				<0.001
First Tertile (Low)	20,928 (39.6)	13,688 (39.9)	7,239 (39.0)	
Second Tertile (Medium)	15,835 (29.9)	11,970 (34.9)	3,864 (20.8)	
Third Tertile (High)	16,144 (30.5)	8,666 (25.3)	7,479 (40.3)	

P-Value: Pearson chi-square test statistic.

GGHE-D: Domestic General Government Health Expenditure as a percentage of gross domestic product (GDP)

### Fixed effects (Measures of association)

We constructed five models (Table 3). We found that men who had primary education (OR: 1.41; 95% CrI 1.31 to 1.52), secondary education (OR: 2.22; 95% CrI 2.05 to 2.41) and tertiary education (OR: 4.40; 95% CrI 3.91 to 4.96) were increasingly more likely to have had an HIV test compared to those with no form of education. We also found that men residents in relatively well-to-do households were increasingly more likely to have been tested compared to men residents in the poorest households: poorer (OR: 1.11; 95% CrI 1.02 to 1.21), middle (OR: 1.15; 95% CrI 1.05 to 1.26), richer (OR: 1.17; 95% CrI 1.06 to 1.30) and richest (OR: 1.31; 95% CrI 1.17 to 1.47) households. Our analysis showed that for every one-year increase in a man’s age, the odds of ever receiving an HIV test increased by 1.03 times (OR: 1.03; 95% CrI 1.03 to 1.03). The odds of having ever received an HIV test increased 1.40 times (OR: 1.40; 95% CrI 1.30 to 1.51) among men in employment compared to unemployed men; and among married/in a union men 2.11 times (OR: 2.11; 95% CrI 1.94 to 2.30) and separated/divorced/widowed men 1.52 times (OR: 1.52; 95% CrI 1.32 to 1.77) compared to men who had never been married nor been in a union.

**Table 3:** Individual, neighborhood and country-level factors associated with the HIV testing among men in Sub-Saharan Africa identified by multilevel logistic regression models.

	Model 1	Model 2	Model 3	Model 4	Model 5
		OR (95% Crl)	OR (95% Crl)	OR (95% Crl)	OR (95% Crl)
**Individual Level Factors**					
**Age (in years)**		1.03*** [1.03,1.03]			1.03*** [1.03,1.03]
**Education (No Education as ref)**					
Primary		1.44*** [1.34,1.55]			1.41*** [1.31,1.52]
Secondary		2.28*** [2.11,2.46]			2.22*** [2.05,2.41]
Higher		4.52*** [4.02,5.07]			4.40*** [3.91,4.96]
**Wealth Index (Poorest as ref)**					
Poorer		1.13** [1.04,1.23]			1.11* [1.02,1.21]
Middle		1.21*** [1.11,1.32]			1.15** [1.05,1.26]
Richer		1.26*** [1.15,1.38]			1.17** [1.06,1.30]
Richest		1.44*** [1.30,1.59]			1.31*** [1.17,1.47]
**Occupation (Not Working as ref)**					
Working		1.40*** [1.30,1.51]			1.40*** [1.30,1.51]
**Marital Status (Never Married as ref)**					
Married/Living together		2.11*** [1.94,2.30]			2.11*** [1.94,2.30]
Divorced/Separated/Widowed		1.53*** [1.32,1.76]			1.52*** [1.32,1.77]
**Internet Usage (Never as ref)**					
Yes, last 12 months		1.82*** [1.70,1.95]			1.80*** [1.69,1.93]
Yes, before last 12 months		1.55*** [1.36,1.78]			1.55** [1.35,1.77]
**Health Insurance Coverage (No as ref)**					
Yes		1.58*** [1.44,1.72]			1.58*** [1.45,1.73]
**Recent Sexual activity (Never had sex as ref)**					
Active in last 4 weeks		1.87*** [1.69,2.07]			1.87*** [1.69,2.08]
Not active in last 4 weeks		1.94*** [1.77, 2.12]			1.93*** [1.76,2.12]
**Exposure to Mass Media (No weekly access to any media as ref)**					
Weekly access to at least one media		1.24*** [1.17,1.32]			1.24*** [1.16,1.31]
**Discriminatory Attitudes (No Discriminatory Attitudes as ref)**					
Discriminatory Attitudes		0.68*** [0.64,0.72]			0.68*** [0.64,0.72]
**HIV Knowledge (Lack comprehensive HIV knowledge as ref)**					
Comprehensive HIV Knowledge		1.41*** [1.33,1.49]			1.40*** [1.32,1.49]
**Risky Sexual Behaviour (No risky sexual behavior as ref)**					
Risky Sexual Behaviour		1.39*** [1.30,1.49]			1.39*** [1.29,1.49]
**Community-Level factors**					
**Community-level poverty**			0.66*** [0.62,0.71]		1.00 [0.92,1.09]
**Community-level Illiteracy**			0.63*** [0.59,0.67]		0.87*** [0.81,0.94]
**Community-Level Unemployment**			0.92** [0.86,0.98]		0.97 [0.90,1.04]
**Community-level Rurality**			0.56*** [0.50,0.64]		0.76*** [0.66,0.86]
**Country-Level factors**					
**Human Development Index (Low as ref)**					
Medium				1.96 [0.60,6.39]	1.58 [0.05,55.09]
**HIV Prevalence (First Tertile [Low] as ref)**					
Second Tertile (Medium)				0.58 [0.28,1.22]	0.48 [0.08,2.96]
Third Tertile (High)				3.08 [0.83,11.47]	2.39 [0.26,22.05]
**GGHE-D(First Tertile [Low] (as ref)**					
Second Tertile (Medium)				0.88 [0.16,4.85]	0.78 [0.20,3.01]
Third Tertile (High)				1.69 [0.63,4.54]	0.98 [0.15,6,40]
**Country-level**					
Variance (95% CrI)	3.85 [0.79,18.86]	4.14 [0.63,27.37]	4.71 [0.68,32.90]	2.24 [0.76,6.62]	4.46 [0.47,41.99]
VPC (%, 95% CrI)	42.1 [13.2,77.7]	45.3 [11.4,84.3]	48.5 [12.2,86.6]	29.7 [12.8,55.0]	47.2 [8.7,89.2]
MOR (95% CrI)	6.50 [2.33,62.96]	6.96 [2.13,146.98]	7.93 [2.20,237.76]	4.17 [2.30,11.64]	7.50 [1.92,483.51]
Explained variation (%)	reference	-7.5 [20.3, -45.1]	-22.3 [13.9, -74.4]	41.8 [3.8,64.9]	-15.8 [40.5, -122.6]
**Community-level**					
Variance (95% CrI)	2.00*** [1.88,2.12]	1.70*** [1.61,1.79]	1.70*** [1.61,1.78]	2.00*** [1.88,2.12]	1.70*** [1.61,1.79]
VPC (%, 95% CrI)	64.0 [44.8,86.4]	63.9 [40.5,89.9]	66.1 [41.0,91.3]	56.3 [44.5,72.6]	65.2 [38.7,93.0]
MOR (95% CrI)	3.85 [3.70,4.01]	3.47 [3.35,3.58]	3.47 [3.35,3.57]	3.85 [3.70,4.01]	3.47 [3.35,3.58]
Explained variation (%)	reference	15.0 [14.4,15.6]	15.0 [14.4,16.0]	0 [0,0]	15.0 [14.4,15.6]
**Model fit statistics**					
DIC	56,988	48,125	56,637	56,987	48,097
**Sample Size**					
Country-level	10	10	10	10	10
Neighborhood-level	4,587	4,559	4,587	4,587	4,559
Individual-level	52,641	49,725	52,641	52,641	49,725

Exponential coefficients; 95% credible intervals in brackets

*P<0.005

**P<0.01

***P<0.001

Model 1: A null model with no explanatory factors to ascertain the variation in the likelihood of HIV testing between communities and countries

Model 2: A model with only individual-level variables

Model 3: A model with only community-level variables

Model 4: A model with only country-level variables

Model 5: An all-inclusive model with individual, community and country-level variables

Compared to men who did not have weekly access to at least one form of media, those with weekly access were 1.24 times (OR: 1.24; 95% CrI 1.16 to 1.31) more likely to have been tested. Similarly, those who had accessed the internet in the last 12 months preceding the survey and had accessed 12 months prior to the survey were 1.80 (OR: 1.80; 95% CrI 1.69 to 1.93) and 1.55 times (OR: 1.55; 95% CrI 1.35 to 1.77) more likely to have been tested than those had never accessed the internet. In addition, possession of health insurance increased the odds of HIV testing by 1.58 times (OR: 1.58; 95% CrI 1.45 to 1.73).

Comprehensive HIV knowledge increased the odds of HIV testing by 1.40 times (OR: 1.40; 95% CrI 1.32 to 1.49), while discriminatory HIV attitudes decreased the odds of testing by 32% (OR: 0.68; 95% CrI 0.64 to 0.72). Compared to those who have never had sex, men who had been sexually active 4 weeks preceding the survey and those who were not were 1.87 (OR: 1.87; 95% CrI 1.69 to 2.08) and 1.93 (OR: 1.93; 95% CrI 1.76 to 2.12) times more likely to have ever received an HIV test respectively. Likewise, men with risky sexual behavior were also 1.39 times (OR: 1.39; 95% CrI 1.29 to 1.49) more likely to have been tested for HIV.

At the community level, men living in communities with high rurality had 24% (OR: 0.76; 95% CrI 0.66 to 0.86) reduced the odds of having had an HIV test, and those living in communities with high illiteracy levels also had 13% (OR: 0.87; 95% CrI 0.81 to 0.94) reduced the odds of having had an HIV test. No statistically significant relationship was found with country-level variables.

### Random effects (Measures of variation)

The variation in the odds of HIV testing across the communities and countries included in this analysis is shown in Table 3 below. We did not find any statistically significant variation in the odds of HIV testing at the country level in all models. However, we found a statistically significant variation in the odds of HIV testing at the community level. The variation was σ2 = 2.00 (95% CrI 1.88–2.12) in the empty model and σ2 = 1.70 (95% CrI 1.61–1.79) in the adjusted and all-inclusive model. The intra-community correlation coefficient estimated using the intercept component variance from the empty model found that 64% of the variance in the odds of HIV testing was due to community-level factors. In addition, we found that the variance in the odds of HIV testing across communities from the adjusted and all-inclusive model was 15%. The median odds ratio buttresses the influence of community-level factors on HIV testing. The empty model had a median odds ratio of 3.07 (95% CrI 3.00–3.13) and 3.47 (95% CrI 3.35–3.58) for the adjusted and all-inclusive model. In essence, a man will be 3.47 times more likely to have an HIV test done when he moves to a community with a higher probability of HIV testing.

## Discussion

HIV testing services uptake among men in these ten Sub-Saharan African countries was 35.1%, with a high of 65.5% in Rwanda and a low of 10.2% in Guinea. HIV testing services uptake was more likely in men with increasing age, some form of formal education, employment, ever-married status, and residents in relatively wealthier households. We also found that men who possessed health insurance, had some form of weekly media exposure, and had accessed the internet were more likely to have ever received an HIV test. Men who had comprehensive knowledge about HIV, were sexually active recently, and engaged in risky sexual behaviors were more likely to have undergone HIV testing. In contrast, those who held discriminatory attitudes towards HIV were less likely to have ever been tested. Furthermore, men in communities with high rurality and illiteracy were less likely to receive an HIV test.

Findings from a previous multilevel study on comprehensive HIV knowledge and HIV testing among men in 29 Sub-Saharan African countries were similar to ours on the increased likelihood of HIV testing among older, ever-married, and educated men [46]. They also found that men with comprehensive HIV knowledge, exposure to mass media, risky sexual behavior, and who were residents in relatively wealthier households were more likely to have accessed HIV testing, same as ours [46]. However, unlike our finding on occupation, they posited that those in employment were less likely to have had an HIV test [46]. Comparably, prior multilevel analyses conducted among men and women have also shown similar findings to ours [11, 47]. Our findings on comprehensive HIV knowledge, education, wealth index, risky sexual behavior, rurality, and HIV discriminatory attitudes align with theirs [11, 47]. Likewise, factors such as age, education, comprehensive HIV knowledge, marital status, risky sexual behavior, media exposure, occupation, and community-level rurality and illiteracy have been identified in multilevel studies among women in Sub-Saharan Africa as significant determinants of HIV testing [48, 49]. However, women in the most relatively well-to-do households were found to be less likely to access HIV testing in a previous study, unlike the finding from our study involving men alone [48].

The increased likelihood of HIV testing uptake with age has been attributed to possible limited access to and usage of HIV testing services, low-risk perception, and relatively poorer comprehensive HIV knowledge, particularly among adolescents [31, 50, 51]. The paper by Kranzer et al, 2011 highlighted slightly different results from our finding by noting reduced HIV testing uptake probability among young people alongside much older individuals of both sexes [52]. The tendency of ever married/being in a union men to have accessed HIV testing services has been ascribed to likely changes in behavior as a result of marriage and fatherhood as well as the availability of a support system in the case of a positive status [53, 54].

Our findings about the increased likelihood of HIV testing uptake with education may be explained by access to and utilization of healthcare services, exposure to HIV prevention messages, and an understanding of the advantages of HIV testing and requisite risk perception [50, 51, 55]. In addition, men in employment usually have stable and higher incomes and better access to education, information, and healthcare services. This may explain why those in employment are more likely to have had an HIV test [48, 56]. However, it has been argued in previous studies that men may have difficulty leaving work and accessing care, supporting their finding that men in employment are less likely to have had an HIV test [46, 55].

Several plausible explanations about the increased likelihood of testing amongst men in relatively wealthier households have been mooted. These men usually have little to no financial and geographical barriers to accessing health care services, and have better exposure to relevant HIV information and education materials [49, 57–59]. Moreover, wealth and education are often interrelated and exhibit a synergistic relationship, each amplifying the benefits of the other [60]. Conversely, men in relatively poorer households may have little to no spare time for HIV testing uptake with a daily round-the-clock focus on making ends meet [49, 57–59]. Additionally, Sub-Saharan Africa has been noted to have higher HIV infection rates among wealthier individuals compared to poorer individuals, unlike the global norm, which may also explain the increased likelihood of testing [61]. Similarly, possession of health insurance addresses financial barriers to accessing care. It allows for routine and unimpeded service when needed, improving the chances of receiving an HIV test among men with health insurance [50, 53].

Our study finding on the positive association between exposure to mass media and internet usage with HIV testing uptake has been expatiated on in multiple studies [31, 56, 62–64]. The association between mass media exposure and HIV testing uptake may be explained by possible exposure to correct, consistent, and comprehensive HIV information on prevention, testing, care, and treatment on mass media platforms [31, 56, 62]. The same explanation on exposure to correct, consistent, and comprehensive HIV information on prevention, testing, care, and treatment may suffice for the positive association between HIV testing uptake and internet usage [63, 64].

The increased likelihood of HIV testing with possession of comprehensive HIV knowledge has been established in multiple studies [46–48, 56, 59, 65]. In addition, men with comprehensive HIV knowledge are less likely to hold misconceptions about HIV prevention, care, and treatment continuum, thereby enabling positive preventive attitudinal behaviors [46–48, 56, 59, 65].

Heightened perceived risk of HIV infection has been attributed to the increased likelihood of testing among persons with recent sexual activity [66, 67]. This was also true for men with risky sexual behavior who were more likely to have had an HIV test, unlike men with a low perceived risk of HIV infection due to abstinence, lacking or having trust in a partner [48, 67]. Besides, it has been noted that having a previous negative test result may reinforce risky sexual behaviors [68].

Men with discriminatory HIV attitudes are less likely to have had an HIV test as this is primarily borne out of misconceptions about HIV [67, 69]. Such men dread being at the receiving end of such stigmatizing attitudes and discrimination; hence they are unlikely to access HIV testing services [67, 69]. Conversely, it has been posited that personal experiences of knowing and caring for a PLHIV may lead to behavior change and HIV testing uptake [53].

At the individual level, men who were employed and lived in relatively economically prosperous households were statistically more likely to have undergone an HIV test. However, this finding did not hold true at the neighborhood level. Neighborhoods with high unemployment and poverty rates showed no statistically significant relationship with HIV testing uptake. Similar to our findings at the individual level, men resident in neighborhoods with high illiteracy rates were less likely to have had an HIV test. Plausible reasons have been adduced, such as exposure to better health knowledge and social networks, influencing the uptake of HIV testing services [70, 71]. Furthermore, the reduced likelihood of men in neighborhoods characterized by high rurality rates having undergone an HIV test can be attributed to socioeconomic factors, poor HIV knowledge, and limited availability and accessibility of HIV testing services in rural areas [56]. None of the country-level variables included in our model had a statistically significant relationship.

Our study’s only evidence of geographical clustering was at the neighborhood level. We found that neighborhood-level factors accounted for 64% of the variance in the odds of HIV testing, while the variance in the odds of HIV testing across communities was 15%. This clustering effect highlights the similarity in HIV testing uptake and associated factors among men in the same neighborhoods. Shared contextual factors and peculiarities in the neighborhoods account for these similarities.

### Implications for practice

Policy implications of these findings are profound and manifold. There is a need to amplify interventions targeted at younger men, those with limited education, and residents of rural and impoverished communities. Initiatives fostering comprehensive HIV knowledge, countering discriminatory attitudes, and promoting safe sexual behaviors can substantially elevate testing uptakes. Given their noted influence, education campaigns that utilize mass media and the internet should be prioritized. Policies that enhance access to health insurance can also play a pivotal role in augmenting testing rates. Consideration for context-specific interventions, given the marked disparities in testing uptakes across different countries, is paramount.

Furthermore, structural reforms are required to bridge the accessibility gap in HIV testing, particularly in remote and underprivileged locales. Implementing mobile testing, community outreach programs, and differentiated service delivery models could significantly mitigate these accessibility constraints. Policies aimed at bolstering the integration of HIV testing into broader healthcare and social service frameworks can further eliminate barriers to testing.

Nuanced and targeted policy formulations are essential to address the specific needs of different demographic segments. For instance, adolescents necessitate tailored approaches that integrate HIV education and testing services into educational curriculums and youth-centric programs, ensuring relevance and effectiveness.

In light of the varying national testing uptakes, there’s a pressing need for policies that are adaptable and responsive to each country’s distinct socioeconomic and cultural landscapes. International collaborations and knowledge exchange can facilitate the development of more refined, effective, and context-sensitive policies, drawing from a diverse pool of experiences and insights.

Lastly, ongoing research and data analysis should underpin policy development and implementation, ensuring adaptability and responsiveness to the evolving dynamics and challenges in HIV testing. A holistic approach, encapsulating the multiplicity of factors influencing testing uptakes, promises the most efficacious pathway to augmenting HIV testing and, by extension, stemming the tide of the epidemic across Sub-Saharan Africa.

### Strength and limitations

A significant strength of this study is our use of nationally representative and high-response DHS datasets from the 10 Sub-Saharan countries included in the analysis. These DHS surveys are conducted using a robust methodology and standardized survey tools. In addition, our multilevel analysis represents the ideal statistical method for producing reliable standard error and estimates by considering the hierarchical nature of the DHS datasets.

The DHS HIV testing and associated variables used in this analysis are based on study participants’ self-reported answers with the risk of recall and social desirability bias. Therefore, the preceding represents a fundamental limitation of our study. Furthermore, we defined neighborhoods using the PSU; however, community contexts and peculiarities may not follow the PSU borders. Moreover, with cross-sectional datasets, we cannot ascribe cumulative effects due to our inability to estimate the length of stay of study participants in a particular neighborhood and/or country.

## Conclusion

Individual and community-level factors influence the uptake of HIV testing among Sub-Saharan African men. Individual-level factors include age, marital status, education, employment status, wealth quintile, possession of health insurance, mass media exposure, internet usage, comprehensive HIV knowledge, recent sexual activity, risky sexual behavior, and discriminatory attitudes to HIV. Community-level factors are rurality and illiteracy rates. In addition, there was evidence of geographical clustering in HIV testing uptake among men at the community level, with about two-thirds of the variability attributable to community-level factors. Therefore, HIV testing programs will need to design interventions that ensure equal access to HIV testing services informed by neighborhood socioeconomic conditions, peculiarities, and contexts.

## Supporting information

S1 Data Stata dataset. (DTA)
